# Comparison of recognition of symptom burden in MPN between patient- and physician-reported assessment – an intraindividual analysis by the German Study Group for MPN (GSG-MPN)

**DOI:** 10.1038/s41375-025-02524-7

**Published:** 2025-02-25

**Authors:** Kirsi Manz, Florian H. Heidel, Steffen Koschmieder, Rudolf Schlag, Jörg Lipke, Frank Stegelmann, Martin Griesshammer, Martine Klausmann, Carl Crodel, Andreas Hochhaus, Holger Schulz, Joachim R. Göthert, Haifa Al-Ali, Heiko Becker, Andreas Reiter, Gernot Beutel, Kim Kricheldorf, Tim H. Brümmendorf, Wolfgang Hoffmann, Konstanze Döhner, Susanne Isfort, On behalf of the German Study Group for Myeloproliferative Neoplasms (GSG-MPN), Florian H. Heidel, Steffen Koschmieder, Rudolf Schlag, Jörg Lipke, Frank Stegelmann, Martin Griesshammer, Martine Klausmann, Carl Crodel, Andreas Hochhaus, Holger Schulz, Joachim R. Göthert, Haifa Al-Ali, Heiko Becker, Andreas Reiter, Kim Kricheldorf, Tim H. Brümmendorf, Konstanze Döhner, Susanne Isfort

**Affiliations:** 1 Department of Hematology, Hemostasis, Oncology and Stem Cell Transplantation, Hannover Medical School (MHH), Hannover, Germany; 2 Institute for Community Medicine, University Medicine Greifswald, Greifswald, Germany; 3 Department of Hematology, Oncology, Hemostaseology, and Stem Cell Transplantation, Faculty of Medicine, RWTH Aachen University, and Center for Integrated Oncology Aachen Bonn Cologne Düsseldorf (CIO ABCD), Aachen, Germany; 4 Hämatologisch-Onkologische Schwerpunktpraxis Würzburg, Würzburg, Germany; 5 Gemeinschaftspraxis für Hämatologie und Onkologie (GEFOS) Dortmund, Dortmund, Germany; 6 Department of Internal Medicine III, University Hospital Ulm, Ulm, Germany; 7 University Clinic for Hematology, Oncology, Haemostaseology and Palliative Care, Johannes Wesling Medical Center Minden, University of Bochum, Bochum, Germany; 8 Gemeinschaftspraxis, Aschaffenburg, Aschaffenburg, Germany; 9 Hematology/Oncology, Jena University Hospital; Comprehensive Cancer Center Central Germany – Campus Jena, Jena, Germany; 10 Practice for Clinical Hematology and Oncology, Frechen, Germany; 11 Department of Hematology, West German Cancer Center (WTZ), University Hospital Essen, Essen, Germany; 12 University Hospital Halle, Halle (Saale), Germany; 13 Department of Medicine I, Medical Center – University of Freiburg, Faculty of Medicine, University of Freiburg, Freiburg, Germany; 14 Department of Hematology and Oncology, University Hospital Mannheim, Heidelberg, Germany

**Keywords:** Myeloproliferative disease, Quality of life

## Abstract

Myeloproliferative neoplasms (MPN) are associated with a variety of symptoms that severely impact patients’ quality of life and ability to perform daily activities. Recent studies showed differences in the perception of physician- versus patient-reported symptom burden. However, studies directly comparing patient- and physician-reported ratings are lacking. Here, a retrospective analysis on symptom burden of 3979 MPN patients of the Bioregistry of the German Study Group for MPN was conducted to intra-individually compare physician and patient reports collected at the same time. Cohen’s kappa was calculated to assess the degree of agreement between patient and physician reports. Factors influencing baseline symptom severity were identified using linear regression and adjusted Cox models were calculated to investigate the effect of symptom burden on survival. MPN patients had a high symptom burden, which neither decreased over time nor upon cytoreductive therapy. All symptoms were more frequently reported by patients compared to physicians. Agreement remained low and only slightly improved when considering a higher threshold for patient symptom severity. Patients with severe symptom burden had inferior survival compared to patients with less severe symptoms. Assessment of symptom burden in MPN is therefore insufficient and patient-reported outcome tools need to be implemented into clinical routine.

## Introduction

Myeloproliferative neoplasms (MPN) are clonal, phenotypically inflammatory diseases [1] associated with a plethora of symptoms that severely impact patients’ quality of life and ability to perform daily activities. MPNs are characterized by high symptom prevalence and burden [2–12]. The most common symptoms reported in real life settings include fatigue, pruritus (itching), night sweats, (bone) pain, weight loss, and fever [13–16].

Symptom control is one of the main treatment goals in MPN serving as primary endpoint in most of the trials leading to approval of new compounds in MPN in the last decades [17, 18]. However, the true impact of symptoms on prognosis remains unclear. Nevertheless, impact of symptom burden on patients´ ability to work and follow their social life has been studied before. In a large cohort of MPN patients, up to 60% of patients with myelofibrosis (MF) had to reduce working hours [2]. Previous studies have shown that the perception of symptom burden and its impact differs between physician- and patient-reported documentation [3, 4, 15]. However, these analyses were conducted without matching patient and physician reports intra-individually. The MPN Landmark survey, which included 813 patients and 457 physicians treating MPN patients, found that physicians significantly underestimated the proportion of patients with symptomatic polycythemia vera (PV) or essential thrombocythemia (ET) at the time of diagnosis compared to patient reporting [4]. Similar findings have been reported for MPN patients in Asia [9]. It is unavoidable that there will be discrepancies in the recognition of symptoms, as the symptom is subjective in nature and experienced exclusively by the patient. It is possible to assess the physician’s ability to correctly report the patient’s perception of symptoms.

To date, little is known about the impact of symptom burden on overall survival in patients with MPN [11, 19]. Two studies that examined symptom burden found contrasting results on the association between symptom burden and patients´ survival [11, 19].

Data from a population-based registry that directly includes patient and physician reports of symptoms at the same point in time are suitable for investigating the intra-individual agreement between these. The aim of this analysis was to assess MPN symptoms in patients enrolled in the German Study Group for MPN (GSG-MPN) Bioregistry and to compare physicians’ and patients’ perceptions of symptom burden on an intra-individual basis. A particular focus of the study was the impact of symptom burden on overall survival of the patients.

## Materials and methods

The German Study Group MPN Bioregistry (GSG-MPN Bioregistry) is an observational study of BCR::ABL1-negative MPN patients with over 70 participating centers, including university hospitals, community hospitals, and office-based hematologists/oncologists. Patients with a confirmed BCR::ABL1-negative MPN diagnosis according to the WHO classification of 2008 [20] or 2016 [21] or IWG-MRT criteria [22], who were 18 years of age or older were included in the bioregistry. Patients are being prospectively followed after enrollment into the registry, with annual recording of clinical data, patient-reported outcomes, and collection of biomaterial. Recruitment started in August 2012, and the present analysis is based on the data of 5198 patients from the first registration and annual follow-up up to 8 years, with a data cut-off date of September 5th, 2023.

### Ethics approval and consent to participate

The study was performed in accordance with the Declaration of Helsinki. The bioregistry was approved by the Ethics Committees of the Medical Faculty of RWTH Aachen University (EK 127/12), University Hospital Ulm (100/13), as well as by each local ethics committee of the participating medical centers. All patients included in the bioregistry provided written informed consent prior to inclusion in the registry. Patients deemed unable to provide written informed consent due to neurological or psychological impairment and patients who did not agree to registration were not included in the bioregistry.

### Symptom assessment

Patients’ symptom ratings were assessed using a modified version of the MPN-SAF-TSS form [6] on a scale from 0 (absent/as good as it can be) to 10 (worst-imaginable/as bad as it can be) for each symptom. A severity score of 7 or higher was considered severe, as seen in the literature [2]. Persistent severe symptoms were defined in this study as symptoms reported as severe at baseline and at the first follow-up visit. The physician’s statement of whether a symptom was present was assessed via a dichotomous question (yes or no, with a category for unknown) and obtained from the electronic case report form (eCRF) of the GSG-MPN registry. Six symptoms were reported by both physician and patient at different times during the course of the disease and included in the analysis: fatigue, pruritus (itching), fever, night sweats, weight loss and pain. The physician’s assessment of “pain” was approximated by “bone pain” as reported by the patients.

Physician assessments were available when patients entered the MPN Registry (baseline/registry inclusion) and at up to 8 annual follow-up visits. The following procedure was used to match patient-reported symptoms with physician-reported symptoms: For each physician assessment date, the date of the patient assessment was taken if the patient assessment was within a 3-month interval around the physician assessment date (physician date ±92 days). If there were multiple patient assessments, the one with the smallest time difference from the physician assessment was selected. Using this approach, the maximum time difference between patient and physician assessments was three months.

The prevalence of patient-reported symptoms was calculated using two different thresholds: Prevalence was defined as a symptom score >0 or ≥4.

### Data

The following data were available for analysis: Demographic data such as year of birth, sex, date and type of diagnosis (coded as essential thrombocythemia (ET), polycythemia vera (PV), primary myelofibrosis (PMF), and other/unclassified MPN), history of thrombosis (yes/no), antithrombotic therapy (yes/no), cytoreductive therapy (with hydroxyurea, interferon alpha, *JAK2* inhibitor and/or anagrelide, yes/no with start date), phlebotomy dependency (yes/no), concurrent medical conditions (with diagnosis, yes/no), mutation status (*JAK2*, *CALR*, *MPL*, yes/no). For dichotomous questions (yes or no), the eCRF also included a category for unknown. Age at enrollment and age at first MPN diagnosis were estimated by subtracting the year of birth from the year of enrollment and the year of first MPN diagnosis. As the exact date of birth was not available, both age variables could only be estimated as whole years. For age-specific analysis, age was categorized as follows: 18–35 as “30”, 36–45 as “40”, 46–55 as “50”, 56–65 as “60”, 66–75 as “70”, 76 and older as “80”. Disease duration was calculated as the time from diagnosis to the baseline visit. Physician demographics were not available.

### Statistical analysis

Continuous demographic variables were summarized as mean and standard deviation (SD) or median and interquartile range (IQR), as appropriate. Fisher’s exact test was used to test for association between two or more categorical variables. The non-parametric Mann–Whitney U test was used to test for differences between two continuous variables (e.g., patient-reported symptom scores), because the normality assumption was not met. McNemar’s test was used to compare the prevalence of physician-reported symptoms and patient-reported symptoms. Cohen’s kappa (κ) [23] was calculated to assess the degree of agreement between patient-reported and physician-reported symptom prevalence and was interpreted as follows [24]: κ between 0 and 0.20 was interpreted as no agreement, κ between 0.21 and 0.39 as minimal agreement, κ between 0.40 and 0.59 as weak agreement, κ between 0.60 and 0.79 as moderate agreement, κ between 0.80 and 0.90 as strong agreement, and κ above 0.9 as almost perfect agreement.

Multiple linear regression analysis was performed to determine associations between baseline symptom severity (dependent variable) and the demographic factors of age, sex, history of thrombosis, anticoagulation therapy, cytoreductive therapy, phlebotomies, and diagnosis (independent variables). Models were adjusted for age and sex. An interaction term between age and sex was allowed. However, if the interaction term was not statistically significant, the models were run without it. Cox models for overall survival were estimated with adjustment for age, sex, and disease duration, and hazard ratios (HRs) were reported with their 95% confidence intervals (CIs). All tests were two-tailed without adjustment for multiple comparisons. *P* values < 0.05 were considered statistically significant. All statistical analyses and data visualization were performed with R software, version 4.3.2. [25]. The R codes are potentially available from the corresponding author upon reasonable request.

## Results

### Baseline characteristics

**Fig. 1:**
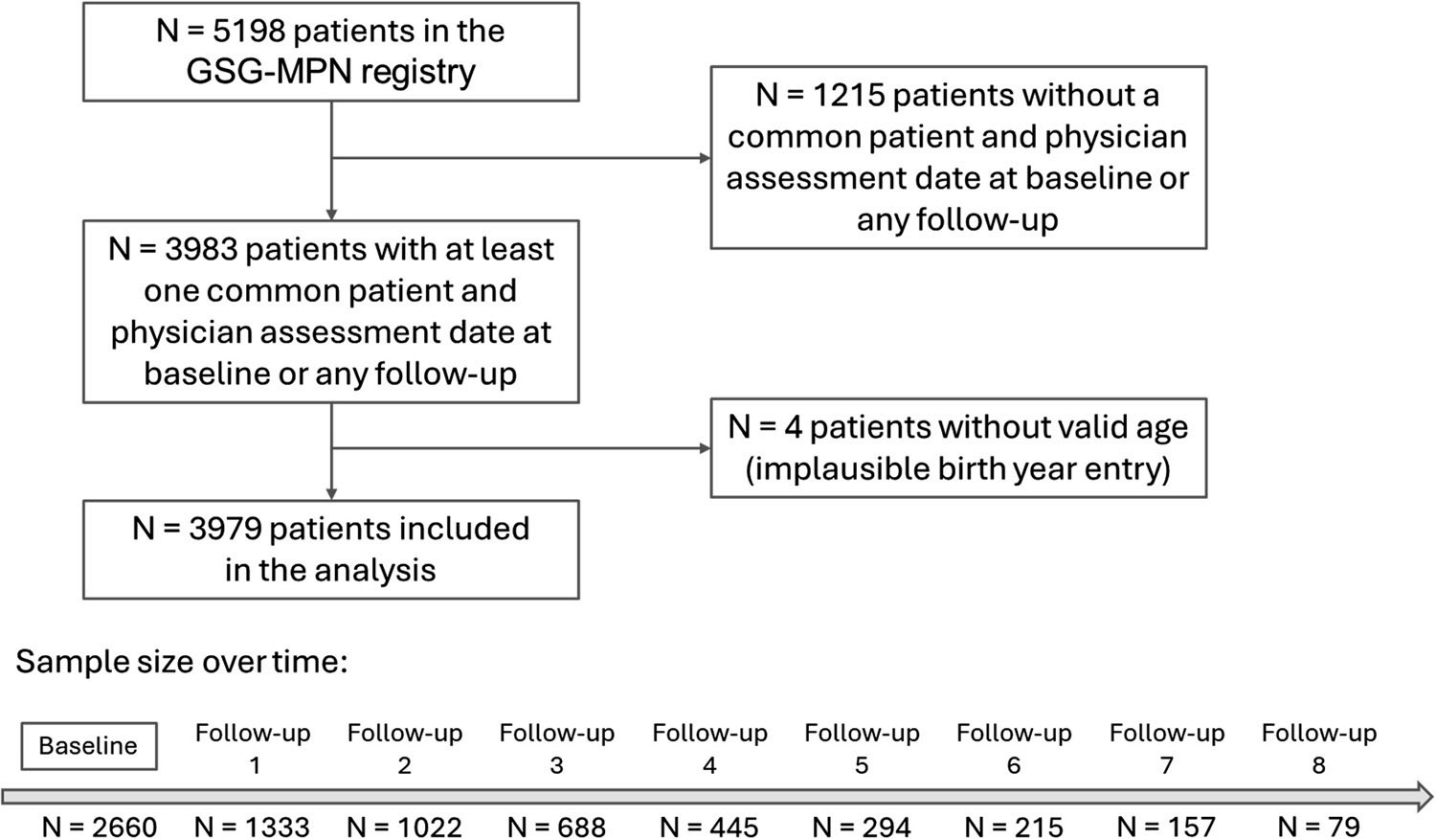
Flow chart of the study. Of all 5198 patients in the registry, a total of 3979 had at least one common assessment date for both patient and physician and were included in the study. Sample size over time refers to the mean number of patients with available patient and physician symptom assessment reports. The exact sample size varies slightly between the six symptoms that were examined.

A total of 3979 patients (76.5% of all GSG-MPN registry patients at the time of data extraction) had at least one common patient-physician symptom assessment at baseline or any follow-up visit and were included in the analysis (see Fig. 1). The sample sizes for the calculations of the symptom prevalence and agreement (baseline) and the symptom prevalence (baseline to follow-up 8) are also shown.

The median age of this cohort was 64 years (range: 18–96), identical to the total of 5198 patients included in the registry. The sample had a similar female-to-male ratio as the overall registry (both 53% female). The diagnosis spectrum was with 38% ET, 33% PV, 21% PMF, and 8% another/unclassified MPN also similar to the overall registry population (38% ET, 32% PV, 21% PMF, and 9% another/unclassified MPN). The characteristics of the included patients are shown in Table 1.

**Table 1:** Patient characteristics.

	Essential thrombocythemia (ET) (*N* = 1526)	Polycythemia vera (PV) (*N* = 1303)	Primary myelofibrosis (PMF) (*N* = 848)	Other/unclassified MPN (*N* = 302)	Overall (*N* = 3 979)
Sex					
Female	933 (61.1%)	619 (47.5%)	390 (46.0%)	153 (50.7%)	2095 (52.7%)
Male	593 (38.9%)	684 (52.5%)	458 (54.0%)	149 (49.3%)	1884 (47.3%)
Age at registry inclusion (years)					
Mean (SD)	60.1 (15.5)	63.4 (13.4)	63.7 (13.1)	64.4 (14.0)	62.3 (14.3)
≥60 y	837 (54.8%)	834 (64.0%)	545 (64.3%)	207 (68.5%)	2423 (60.9%)
≥65 y	660 (43.3%)	656 (50.3%)	437 (51.5%)	172 (57.0%)	1925 (48.4%)
Age at first diagnosis (years)					
Mean (SD)	53.9 (16.3)	57.3 (14.4)	59.5 (13.6)	60.8 (14.3)	56.7 (15.2)
Disease duration (years)					
Median (IQR)	3.7 (0.8–9.5)	4.3 (0.8–9.6)	2.0 (0.4–5.9)	1.4 (0.2–5.2)	3.3 (0.6–8.4)
History of thrombosis	424 (27.8%)	422 (32.4%)	202 (23.8%)	74 (24.5%)	1122 (28.2%)
History of bleeding event(s)	41 (2.7%)	41 (3.1%)	32 (3.8%)	11 (3.6%)	125 (3.1%)
Antithrombotic therapy^a^	1073 (70.3%)	1030 (79.0%)	453 (53.4%)	156 (51.7%)	2712 (68.2%)
Cytoreductive therapy	1055 (69.1%)	846 (64.9%)	525 (61.9%)	149 (49.3%)	2575 (64.7%)
Duration of cytoreductive therapy (years)					
Median (IQR)	3.0 (0.5–7.5)	2.6 (0.4–6.5)	1.6 (0.3–4.7)	1.6 (0.1–6.6)	2.4 (0.4–6.5)
Phlebotomies	81 (5.3%)	879 (67.5%)	60 (7.1%)	30 (9.9%)	1050 (26.4%)
*JAK2* mutation	920 (60.3%)	1 140 (87.5%)	500 (59.0%)	161 (53.3%)	2721 (68.4%)
*CALR* mutation	259 (17.0%)	4 (0.3%)	146 (17.2%)	13 (4.3%)	422 (10.6%)
*MPL* mutation	47 (3.1%)	4 (0.3%)	39 (4.6%)	4 (1.3%)	94 (2.4%)

All data is shown at the time of registry inclusion unless otherwise noted.

*SD* standard deviation, *IQR* interquartile range.

^a^Including anticoagulants and anti-platelet agents.

### Patient and physician perspectives on baseline symptom prevalence

**Fig. 2:**
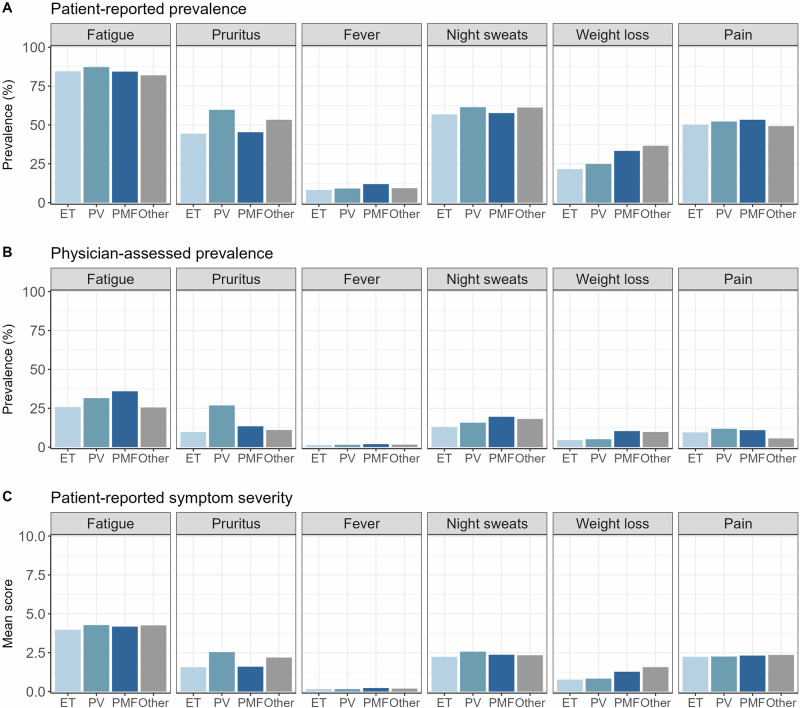
Symptoms at baseline. Patient-reported prevalence (**A**), physician-assessed prevalence (**B**), and mean patient-reported symptom severity (**C**) at baseline are shown. Prevalence was defined as a symptom score greater than or equal to 1 (patient) or “yes” (physician). ET essential thrombocythemia, PV polycythaemia vera, PMF primary myelofibrosis, other other/unclassified MPN.

Overall, 93% of patients of this cohort reported the presence of at least one symptom at baseline (ET: 93%, PV: 95%, PMF: 92%, other/unclassified MPN: 94%). Severe disease burden (i.e. any symptom score ≥7) was present in 38% of the patients (ET: 34%, PV: 42%, PMF: 37%, other/unclassified MPN: 44%). Baseline patient and physician symptom prevalence and mean symptom severity scores are shown in Fig. 2.

Fatigue was the most common symptom reported by both patients and physicians. It was reported by 85%, 82%, 84%, and 82% of patients with ET, PV, PMF, and other/unclassified MPN, respectively. These differences between diagnostic groups were not statistically significant. Physician assessment of the same patients showed statistically significantly different prevalences ranging from 26% (ET and other/unclassified MPN) to 36% (PMF; *p *< 0.001). Night sweats, pain, and pruritus were the second most common symptoms, followed by weight loss and fever. Pruritus was reported by both patients and physicians as having the highest prevalence in patients with PV (60% and 27%, respectively). This difference between MPN entities was statistically significant for both patient and physician reporting (*p *< 0.001). Another statistically significant difference was seen for weight loss, the prevalence of which was the lowest in patients with ET and the highest in patients with unclassified/other MPN.

### Physician and patient agreement on baseline symptom prevalence

Agreement on baseline symptom prevalence between physician and patients´ assessment was quite low. Even for symptoms which are objectively measurable, such as weight loss, reports on symptom frequency were discrepant (any (unintentional) weight loss in the past 6 months reported by 26% (706/2687) of patients, whereas physicians reported weight loss in only 6% (169/2687) of patients). Table 2 shows physician and patient ratings of weight loss at baseline for the 2687 patients for whom both physician and patient ratings were available.

**Table 2:** Cross tabulation of weight loss as assessed by physician and patient.

Weight loss		Patient			
		No	Score 1–10	Score 4–10	Score 7–10
Physician	Yes	44	125	85	45
	No	1937	581	194	75
	Total	1981	706	279	120

In 44 cases the physician reported weight loss while the patient did not indicate burden from this symptom.

Similar cross-tabulations were used to calculate the prevalence of symptoms in patients with and without cytoreductive therapy, as well as the agreement κ between physician and patient assessment (see Table 3). In all but two cases, there was a statistically significant difference in agreement between physician and patient with *p *< 0.05, except for fever with a prevalence assumption of 4 or more points for both patients with and without cytoreductive therapy, where there was no statistically significant difference in agreement. For all symptoms, patients reported a higher prevalence of symptoms than physicians. Agreement was low and improved only slightly for symptoms with a severity score of ≥4. In addition, agreement was similar for patients with and without cytoreductive therapy.

**Table 3:** Symptom prevalence perception at baseline according to physician and patient, and agreement between them.

		Baseline prevalence 1–10 points^a^			Baseline prevalence 4–10 points^b^		
Symptom	Physician reported^c^ (%, *n*)	Patient reported (%, *n*)	Level of agreement	Physician reported vs. patient reported (κ, 95% CI)	Patient reported (%, *n*)	Level of agreement	Physician reported vs. patient reported (κ, 95% CI)
Patients with cytoreductive therapy							
Fatigue	29.8% (525/1761)	86.4% (1522/1761)	None	0.11 (0.09, 0.13)	56.0% (986/1761)	Minimal	0.23 (0.20, 0.28)
Pruritus	16.1% (281/1743)	49.9% (869/1743)	Minimal	0.28 (0.25, 0.31)	22.5% (392/1743)	Minimal	0.34 (0.28, 0.39)
Fever	1.7% (30/1759)	9.5% (167/1759)	None	0.05 (0.00, 0.11)	1.1% (19/1759)	None	0.11 (−0.02, 0.24)
Night sweats	15.6% (279/1783)	60.0% (1069/1783)	None	0.18 (0.15, 0.21)	28.4% (507/1783)	Minimal	0.30 (0.25, 0.35)
Weight loss	6.6% (115/1753)	27.6% (484/1753)	None	0.20 (0.16, 0.24)	11.1% (194/1753)	Minimal	0.32 (0.25, 0.39)
Pain	10.6% (173/1636)	53.8% (800/1636)	None	0.13 (0.10, 0.16)	29.0% (475/1636)	None	0.19 (0.14, 0.24)
Patients without cytoreductive therapy							
Fatigue	30.1% (281/933)	82.7% (772/933)	None	0.14 (0.12, 0.17)	49.8% (465/933)	Minimal	0.36 (0.31, 0.42)
Pruritus	17.0% (157/921)	51.6% (475/921)	Minimal	0.30 (0.26, 0.35)	20.2% (186/921)	Minimal	0.37 (0.30, 0.45)
Fever	1.2% (11/919)	9.1% (84/919)	None	0.11 (0.02, 0.19)	1.4% (13/919)	Weak	0.41 (0.16, 0.66)
Night sweats	15.8% (148/939)	56.7% (532/939)	Minimal	0.22 (0.18, 0.26)	26.6% (250/939)	Minimal	0.37 (0.30, 0.44)
Weight loss	5.8% (54/934)	23.8% (222/934)	Minimal	0.22 (0.15, 0.28)	9.1% (85/934)	Minimal	0.34 (0.23, 0.45)
Pain	9.8% (86/881)	47.1% (415/881)	None	0.15 (0.11, 0.20)	23.8% (210/881)	Minimal	0.27 (0.20, 0.34)

Level of agreement reported according to McHugh [24].

*κ* Cohen’s kappa, *CI* confidence interval.

^a^Prevalence was defined as a symptom score greater than or equal to 1, as rated on the questionnaire.

^b^Prevalence was defined as a symptom score greater than or equal to 4, as rated on the questionnaire.

^c^Prevalence was defined as present or absent, as recorded in the medical documentation by the physician.

**Fig. 3:**
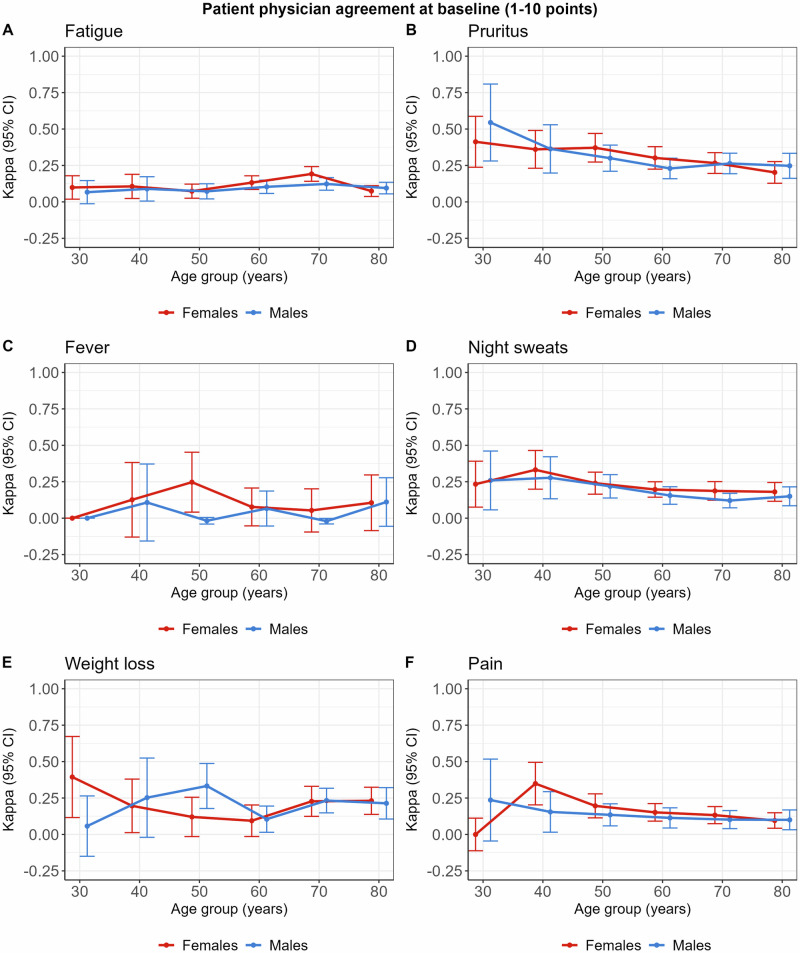
Agreement between patient and physician at baseline. Agreement regarding symptom presence of fatigue (**A**), pruritus (**B**), fever (**C**), night sweats (**D**), weight loss (**E**) and pain (**F**). Kappa Cohen’s kappa, CI confidence interval. Agreement is shown for female and male MPN patients across age groups.

Age and sex dependency of patient-physician agreement is shown in Fig. 3. No clear difference in agreement could be detected for fatigue, fever, and weight loss for age or between male and female patients. Regarding pruritus, night sweats, and pain, agreement decreased slightly with increasing age, similarly for both male and female patients. Overall, there is no clear difference between male and female MPN patients in agreement about the presence of symptoms as rated by both patients and physicians. Focusing on agreement analysis when patients reported symptom severity of 4 or more points did not provide any additional insight.

### Factors influencing baseline symptom severity

Linear regression analyses for symptom severity (see Supplementary Table 1) showed some statistically significant associations with demographic variables, but most associations appeared rather subtle. Considering changes of approximately one score point as relevant associations, the following associations with pruritus remained: Pruritus was 0.92 (CI: 0.75, 1.22) points higher in PV patients than in ET patients, and 0.85 (CI: 0.63, 1.08) points higher in phlebotomy-dependent patients. In addition, with a decrease in fatigue of 0.23 points per 10 years of age, patients in their twenties had approximately 1.2 points higher fatigue scores than patients in their seventies. When combined with sex (women had a 0.51-point higher fatigue score than men), fatigue scores were the highest in young female patients.

### Symptom prevalence over time

**Fig. 4:**
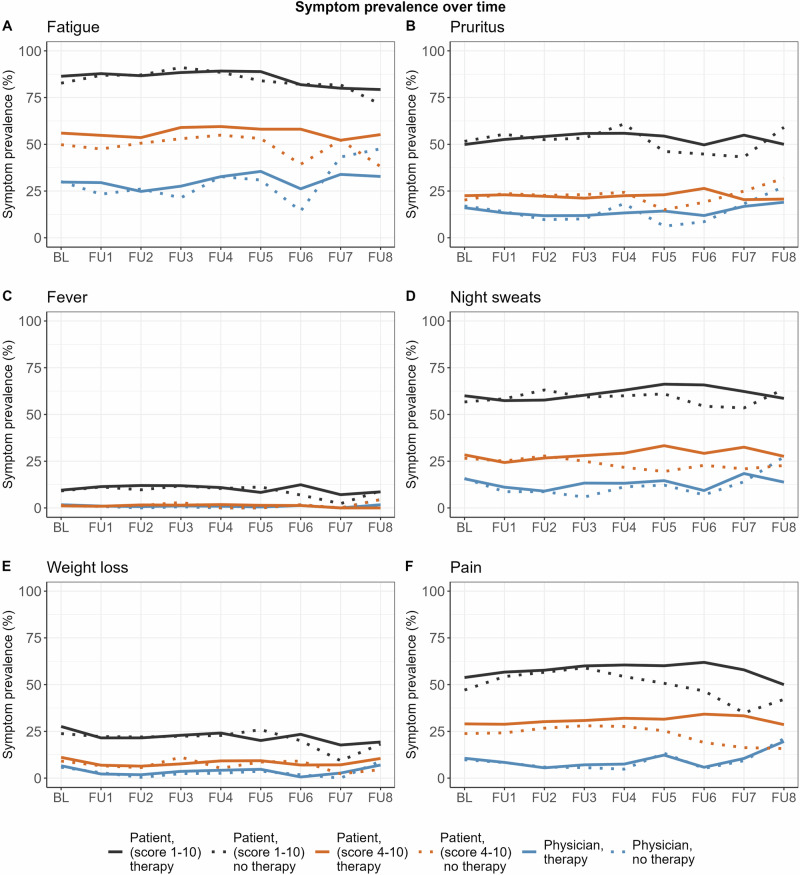
Prevalence of symptoms over time. Prevalence is shown for baseline (BL) and annual follow-up (FU) visits for fatigue (**A**), pruritus (**B**), fever (**C**), night sweats (**D**), weight loss (**E**) and pain (**F**). Black lines show prevalence calculated from patient-reported symptom severity, where prevalence was defined as a symptom score greater than or equal to 1. Brown lines show prevalence calculated from patient-reported symptom severity, where prevalence was defined as a symptom score greater than or equal to 4. Blue lines show physician-reported prevalence. Solid lines represent patients with cytoreductive therapy and dotted lines represent patients without cytoreductive therapy. For brevity, the legend uses the word “therapy” instead of cytoreductive therapy.

Symptom prevalence over time calculated from patients and physicians ratings is shown in Fig. 4. Patient-reported symptom prevalence was remarkably higher than the prevalence perceived by the physician. This was consistent for all symptoms. Symptom prevalence was not lower in patients who received cytoreductive therapy. There was no trend towards better physician symptom recognition over time. Of note, sample size for the later follow-up visits significantly decreased compared to baseline (see Fig. 1 for numbers).

### Patient-reported severe symptom burden and overall survival

**Fig. 5:**
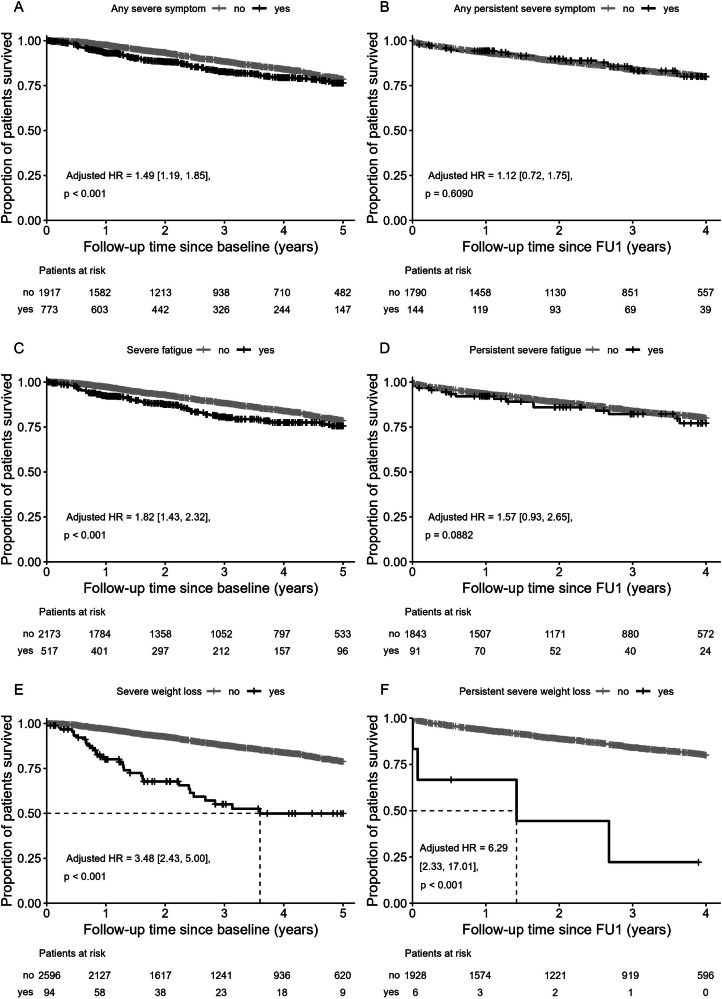
Overall survival and symptoms. Overall survival by the presence of at least one severe symptom at baseline (**A**), at least one persistent severe symptom (**B**), severe fatigue at baseline (**C**), persistent severe fatigue (**D**), severe weight loss at baseline (**E**), and persistent severe weight loss (**F**). Persistent severe symptoms were symptoms reported as severe at baseline and at the first follow-up visit (FU1). Graphs on the right exclude patients who died or were censored between the baseline and FU1 visit.

No statistically significant difference in the presence of severe symptom burden at baseline between patients receiving cytoreductive therapy and those without could be detected (Supplementary Table 2). Likewise, the persistence of severe symptoms did not differ between patients with and without cytoreductive therapy. The presence of any severe symptom at baseline (Fig. 5A) was associated with a higher hazard of death in a multivariable model adjusted for age, sex, and disease duration (adjusted HR = 1.5 (CI: 1.2, 1.9), *p *< 0.001). Among the six symptoms, severe fatigue at baseline (Fig. 5C) and severe weight loss at baseline (Fig. 5E) were both associated with increased hazard of dying, with adjusted HR = 1.8 (CI: 1.4, 2.3), *p *< 0.001 and adjusted HR = 3.5 (CI: 2.4, 5.0), *p *< 0.001, respectively. Considering symptom persistence resulted in a non-significant hazard of death (Fig. 5B). Persistent fatigue (Fig. 5E) did not result in a significantly higher hazard ratio. Despite low numbers of patients with persistent weight loss, a higher risk could be confirmed (Fig. 5F, adjusted HR = 6.3 (CI: 2.3, 17.0), *p *< 0.001).

In order to exclude the possibility that the cause of death may influence the correlation, a subgroup of 195 patients was analyzed in which the cause of death was reported. Notably, symptom presence and burden did not differ between patients who died due to MPN and those who died due to unrelated causes (Supplementary Table 3).

## Discussion

The present study retrospectively analyzed the symptom burden of patients with Myeloproliferative Neoplasms (MPN), specifically Essential Thrombocythemia (ET), Polycythemia vera (PV), Primary Myelofibrosis (PMF), and other/unclassified MPNs, focusing on six key symptoms. At baseline, 93% of the patients were symptomatic, consistent with other studies reporting similarly high symptom prevalence [2–12]. Severe symptom burden, defined as a score of 7–10 in at least one symptom, was present in 38% of patients. The median disease duration at baseline was 3.3 years. Fatigue was the most frequently reported symptom by both patients and physicians, followed by night sweats, pain, pruritus, weight loss, and fever, mirroring patterns seen in other studies [2, 5, 6, 8–11]. Pruritus was particularly prevalent in PV patients, reported by both patients (60%) and physicians (27%).

In terms of symptom burden, PMF patients did not exhibit an increased burden compared to those with ET, PV, and other/unclassified MPNs, which aligns with previous studies [10, 12, 26]. The only exception was pain showing a slightly higher prevalence in PMF patients (53%), compared to the other entities (ET: 50%, PV: 52%, other/unclassified MPN: 49%). Overall, the disease burden in PMF patients in our cohort appeared lower compared to other published cohorts.

Symptom burden, as reported by both patients and physicians, did not decrease over time, consistent with findings from other studies [9, 19, 27]. Patients receiving cytoreductive therapy reported similar symptom burdens to those not receiving such therapy. Cytoreductive treatments included commonly used drugs such as hydroxycarbamide, anagrelide, interferon and JAK inhibitors, as well as less frequently used drugs like busulfan or experimental treatments. Unfortunately, the registry did not provide detailed treatment information to perform subgroup analysis. Unexpectedly, symptom burden, with or without treatment, remained unchanged over time. Physicians also reported no difference in symptom burden between patients with and without cytoreductive treatment. Similar findings, or even deterioration of symptoms following cytoreductive treatment, have been reported in other studies [9, 12, 19]. In recent reports on ET and PV patients, improvement in symptom burden was mainly observed in those with high symptom burden at initial diagnosis, while worsening was noted in patients with low baseline symptom burden following cytoreductive treatment [28]. Due to the lack of detailed treatment information, we can only hypothesize that the beneficial effects of symptom-oriented treatment, especially JAK inhibitors, might have been obscured due to the small sample size in our cohort.

Patient-physician consensus on the presence of symptoms was low, improving only slightly for symptoms with a severity score of ≥4. Fever in patients not receiving cytoreductive therapy exhibited the highest patient-physician consensus, although this agreement was still weak. We found only one study with data on simultaneous patient-physician symptom assessment in MPN patients, which similarly showed that patients reported higher symptom presence than physicians [9]. The MPN Landmark survey indicated that physicians underestimated the proportion of patients with symptomatic PV or ET at diagnosis compared to patient reports [4]. However, physician and patient responses were not matched in that survey. Comparable findings have been observed in metastatic breast cancer patients, where physicians significantly underreported symptoms compared to patients [29], and in multiple myeloma patients, where there was poor to fair agreement between patients and physicians in reporting treatment side effects [30]. Furthermore, the discrepancies in symptom perception between patients and physicians did not diminish over time, which is consistent with the literature [31].

Our results confirm that physicians tend to underestimate the presence of symptoms in their patients compared to patient reports. Several factors could contribute to this discrepancy: Patients might prefer to report symptoms via questionnaires rather than during visits, possibly omitting symptoms they consider less relevant in the communication with their physicians. Physicians may also fail to recognize symptoms that are not easily measurable. The patient and physician may also record the patient’s symptoms for different reasons: The patient is encouraged to record the symptoms so that the physician using the questionnaire is properly informed about the patient’s condition. The physician records symptoms as a useful decision-making tool for prognostic stratification of the patient. A systematic review comparing patient-reported and clinician-observed symptoms in cancer patients found that physician assessments were more aligned with clinical outcomes, whereas patients reported symptoms impacting their daily activities and quality of life [31]. The MPN Landmark survey also found that only 26% of participating physicians used a validated symptom assessment form, with nearly half using their own rating methods [3].

Most notably, we found a significant association between overall survival and the presence of severe symptoms, primarily due to severe fatigue and severe weight loss. After considering persistent severe symptom burden (patient-reported score of 7 or higher at baseline and first follow-up), the hazard of death decreased. These results suggest that severe symptoms may serve as a predictor of short-term survival in MPN patients. However, we still observed a significant effect of persistent severe weight loss on overall survival despite the small number of patients with persistent severe weight loss.

In a study on Thai MPN patients, symptom burden was not associated with inferior overall survival [11], although it included a lower number (*n* = 80) of patients and identified only the strongest predictors as significantly associated with survival. Conversely, a recent study including approximately 800 participants from a Canadian MPN registry found a significantly higher risk of death in patients with higher symptom burden, defined as >20 points on the MPN-SAF Total Symptom Score [19]. Therefore, the impact of symptom burden on patient survival warrants further investigation in future studies.

The presence of constitutional symptoms is included in prognostic scores for MF, such as IPSS [32], DIPSS [33], DIPPS+ [34] and MIPSS70 [35], but to our knowledge not in prognostic scores for ET or PV. Given our findings on the effect of severe symptom burden on overall survival, the introduction of new prognostic scores incorporating a symptom component may be justified in the future.

The main strength of the study is the availability of both patient and physician reports, documented at approximately the same point in time. Despite patients reporting on a scale of 0–10 and physicians reporting as yes or no, we were able to examine the agreement on the presence of symptoms from both perspectives. Furthermore, this study benefits from its large sample size with data from a population-based registry of 3979 MPN patients. The longitudinal design allowed us to examine symptom burden over time. Our analysis included 77% of all patients enrolled in the GSG-MPN Bioregistry, and our cohort´s characteristics are similar to those of the overall registry population, excluding a selection bias. Moreover, the GSG-MPN Bioregistry is representative of the German MPN patient population.

A major limitation of the study is related to the sample size: although high numbers were available for baseline, lower numbers were available for the follow-up visits (with only 80 patients at the final data point). Within the Bioregistry of the German MPN Study Group more than 6000 MPN patients have been registered so far. Missing patient questionnaires were the main reason for not including all patients in our analysis. At baseline, physician assessments were missing in 3.2% (166/5198) of cases. In order to include as many data points as possible, we also incorporated partially completed questionnaires and assessments. A second limitation of our study was the interval of assessments: within the registry documentation is scheduled in yearly intervals. Therefore, short-term changes in symptom burden and/or severity could not be assessed at higher temporal granularity. However, when considering the complete cohort, symptom prevalence appeared to be rather stable over time. In general, symptom burden in MPN patients was high and did not decrease over time. Discrepancy in symptom recognition between patients and physicians was evident, with physicians underestimating symptom burden as reported by their patients. Of note, patient-reported symptom burden was not lower in those on cytoreductive therapy, and physicians did not report symptoms differently when their patient was receiving cytoreductive treatment. A third limitation of our study was the fact that physicians did not rate symptoms on the same questionnaire as the patients, but, for physicians´ assessments, the mere presence or absence of a symptom was documented from the medical record.

Taken together, this analysis in a large cohort of MPN patients suggests significant discrepancies in symptom recognition in an intra-individual assessment between patient-reported and physician-reported data. Physicians reported a much lower symptom presence than patients. Severe patient-reported symptoms were found to be an independent risk factor for short-term death and should be carefully assessed in routine clinical practice. These findings highlight the need to include patient-reported outcome tools, such as validated symptom questionnaires, into both clinical routine and the drug development process in clinical trials [36].

## Supplementary information

Supplementary Information

## Data Availability

The datasets generated during and/or analyzed during the current study are potentially available from the corresponding author on reasonable request.
