# Supplementary material for: Comparison of recognition of symptom burden in MPN between patient- and physician-reported assessment – an intraindividual analysis by the German Study Group for MPN (GSG-MPN)
Source: Leukemia. 2025 Feb 25;39(4):864–75. doi: 10.1038/s41375-025-02524-7 (PMC11976279; doi:10.1038/s41375-025-02524-7)
Supplement: Supplementary file 1 — Supplementary Information [file 41375_2025_2524_MOESM1_ESM.docx]

**Supplementary Information**

Supplementary Table 1: Age- and sex-adjusted linear models for baseline symptom severity

| **Characteristic** | **Fatigue**  (β, 95% CI) | **Pruritus**  (β, 95% CI) | **Fever**  (β, 95% CI) | **Night sweats**  (β, 95% CI) | **Weight loss**  (β, 95% CI) | **Pain**  (β, 95% CI) |
| --- | --- | --- | --- | --- | --- | --- |
| **Age**^a^  (per 10 years) | -0.23  (-0.31, -0.16)*** | -0.08  (-0.15, -0.01)* | -0.02  (-0.04, -0.01)* | 0.07  (-0.15, 0.00)* | 0.15  (0.10, 0.20)*** | -0.01  (-0.08, 0.07) |
| **Male sex**^a^  (ref.: female) | -0.51  (-0.72, -0.29)*** | -0.06  (-0.26, 0.14) | 0.07  (0.02, 0.13)* | -0.30  (-0.51, -0.09)** | 0.14  (-0.01, 0.30) | -0.69  (-0.90, 0.48)*** |
| **Diagnosis** (ref.: ET) |  |  |  |  |  |  |
| PV | 0.43  (0.18, 0.69)*** | 0.92  (0.75, 1.22)*** | 0.01  (-0.06, 0.08) | 0.43  (0.17, 0.68)*** | 0.00  (-0.18, 0.19) | 0.15  (-0.10, 0.41) |
| PMF | 0.36  (0.07, 0.64)* | 0.08  (-0.19, 0.35) | 0.07  (-0.01, 0.14) | 0.18  (-0.10, 0.47) | 0.44  (0.23, 0.66)*** | 0.24  (-0.05, 0.53) |
| Other / unclassified MPN | 0.43  (-0.01, 0.88) | 0.59  (0.18, 1.00)** | 0.08  (-0.04, 0.20) | 0.15  (-0.29, 0.58) | 0.65  (0.33, 0.97)*** | 0.10  (-0.34, 0.54) |
| **History of thrombosis** (ref.: no) | 0.33  (0.09, 0.57)** | 0.21  (-0.01, 0.44) | 0.00  (-0.06, 0.07) | -0.08  (-0.31, 0.16) | 0.01  (-0.17, 0.18) | 0.17  (-0.07, 0.41) |
| **Anticoagulation therapy** (ref.: no) | 0.07  (-0.18, 0.31) | 0.03  (-0.20, 0.26) | 0.01  (-0.06, 0.07) | -0.08  (-0.32, 0.16) | -0.18  (-0.35, 0.00) | 0.16  (-0.08, 0.41) |
| **Cytoreductive therapy** (ref.: no) | 0.46  (0.23, 0.69)*** | 0.10  (-0.12, 0.32) | 0.04  (-0.02, 0.10) | 0.18  (-0.05, 0.41) | 0.05  (-0.12, 0.22) | 0.43  (0.19, 0.66)*** |
| **Phlebotomies**  (ref.: no) | 0.23  (-0.01, 0.47) | 0.85  (0.63, 1.08)*** | -0.01  (-0.07, 0.05) | 0.27  (0.03, 0.50)* | -0.27  (-0.45, -0.10)** | 0.06  (-0.18, 0.30) |

^a^ = Univariable models for age and sex are shown. β = regression coefficient, CI = confidence interval, ref. = reference (category), ET = essential thrombocythemia, PV = polycythaemia vera, PMF = primary myelofibrosis, CI = confidence interval, * p < 0.05, ** p < 0.01, *** p < 0.001.

Supplementary Table 2: Comparison of severe symptoms and severe persistent symptoms in MPN patients with and without cytoreductive therapy

|  | **Severe symptoms** | | | **Persistent severe symptoms** | | |
| --- | --- | --- | --- | --- | --- | --- |
| **Symptom** | **Cytoreductive therapy (%, n)** | **No cytoreductive therapy (%, n)** | **P value** | **Cytoreductive therapy (%, n)** | **No cytoreductive therapy (%, n)** | **P value** |
| **Any** | 29.3%  (755/2 575) | 27.8%  (390/1 404) | 0.3219 | 8.8%  (165/1 873) | 7.3%  (85/1 158) | 0.1736 |
| **Fatigue** | 18.8%  (485/2 575) | 18.7%  (262/1 404) | 0.9268 | 5.2%  (97/1 873) | 4.5%  (52/1 158) | 0.4441 |
| **Pruritus** | 7.7%  (198/2 575) | 8.3%  (116/1 404) | 0.5627 | 1.3%  (25/1 873) | 2.3%  (27/1 158) | 0.0562 |
| **Fever** | 0.2%  (6/2 575) | 0.1%  (2/1 404) | 0.7205 | 0.0%  (0/1 873) | 0.0%  (0/1 158) | NA |
| **Night sweats** | 9.3%  (240/2 575) | 10.2%  (143/1 404) | 0.4079 | 2.6%  (49/1 873) | 1.8%  (21/1 158) | 0.1919 |
| **Weight loss** | 3.2%  (83/2 575) | 3.6%  (51/1 404) | 0.5540 | 0.3%  (6/1 873) | 0.5%  (6/1 158) | 0.3920 |
| **Pain** | 10.3%  (266/2 575) | 8.5%  (119/1 404) | 0.0667 | 3.1%  (58/1 873) | 2.0%  (23/1 158) | 0.0844 |

NA = not applicable. Severe symptoms as reported at baseline, persistent severe symptoms present at baseline and first follow-up visit.

Supplementary Table 3: Comparison of symptom presence and burden in 195 patients who died of MPN and of other reasons than MPN.

| **Causes of death** | | | |
| --- | --- | --- | --- |
|  | **MPN (N=104)** | **non-MPN (N=91)** | **P-value** |
| **Male sex** | 62 (59.6%) | 53 (58.2%) | 0.8846 |
| **Age at registry inclusion** (years) | 70.8 (9.36) | 71.0 (9.83) | 0.7726 |
| **Diagnosis** |  |  |  |
| ET | 18 (17.3%) | 21 (23.1%) | **0.0350** |
| PV | 29 (27.9%) | 39 (42.9%) |  |
| PMF | 48 (46.2%) | 26 (28.6%) |  |
| Other | 9 (8.7%) | 5 (5.5%) |  |
| **Disease duration (years)** | 6.16 (7.09) | 6.12 (5.34) | 0.4386 |
| **Any severe symptom** | 38 (36.5%) | 32 (35.2%) | 0.8818 |
| **Severe fatigue** | 28 (26.9%) | 23 (25.3%) | 0.8707 |
| **Severe pruritus** | 6 (5.8%) | 9 (9.9%) | 0.2971 |
| **Severe fever** | 0 (0%) | 0 (0%) | >0.9999 |
| **Severe night sweats** | 5 (4.8%) | 11 (12.1%) | 0.0727 |
| **Severe weight loss** | 15 (14.4%) | 6 (6.6%) | 0.1049 |
| **Severe pain** | 9 (8.7%) | 12 (13.2%) | 0.3587 |
| **Fatigue score** | 4.93 (2.94) | 4.61 (3.13) | 0.5137 |
| **Pruritus score** | 1.94 (2.57) | 2.00 (2.93) | 0.7992 |
| **Fever score** | 0.38 (1.13) | 0.41 (1.32) | 0.4549 |
| **Night sweat score** | 2.32 (2.49) | 2.55 (3.17) | 0.7939 |
| **Weight loss score** | 2.93 (3.38) | 2.03 (3.08) | 0.0711 |
| **Pain score** | 2.47 (2.80) | 2.78 (3.31) | 0.8923 |

Frequency (%) are presented for categorical variables and mean (SD) for continuous variables. Statistically significant p-values are shown in bold.
